# Excellent low-voltage operating flexible ferroelectric organic transistor nonvolatile memory with a sandwiching ultrathin ferroelectric film

**DOI:** 10.1038/s41598-017-09533-2

**Published:** 2017-08-21

**Authors:** Ting Xu, Lanyi Xiang, Meili Xu, Wenfa Xie, Wei Wang

**Affiliations:** State Key Laboratory on Integrated Optoelectronics, College of Electronic Science and Engineering, Jilin University, 2699 Qianjin Street, Changchun, 130012 China

## Abstract

The high operating voltage is a primary issue preventing the commercial application of the ferroelectric organic field-effect transistor (Fe-OFET) nonvolatile memory (NVM). In this work, we propose a novel route to resolve this issue by employing two ultrathin AlO_X_ interfacial layers sandwiching an ultrathin ferroelectric polymer film with a low coercive field, in the fabricated flexible Fe-OFET NVM. The operation voltage of Fe-OFET NVMs decreases with the downscaling thickness of the ferroelectric film. By inserting two ultrathin AlO_X_ interfacial layers at both sides of the ultrathin ferroelectric film, not only the gate leakage is prominently depressed but also the mobility is greatly improved. Excellent memory performances, with large mobility of 1.7 ~ 3.3 cm^2^ V^−1^ s^−1^, high reliable memory switching endurance over 2700 cycles, high stable data storage retention capability over 8 × 10^4^ s with memory on-off ratio larger than 10^2^, are achieved at the low operating voltage of 4 V, which is the lowest value reported to data for all Fe-OFET NVMs. Simultaneously, outstanding mechanical fatigue property with the memory performances maintaining well over 7500 bending cycles at a bending radius of 5.5 mm is also achieved in our flexible FE-OFET NVM.

## Introduction

Ferroelectric organic field-effect transistor (Fe-OFET) based nonvolatile memory (NVM) has attracted considerable attention for its single transistor memory element configuration, short programming time, non-destructive readout and nonvolatile data storage capabilities^1, 2^. In a Fe-OFET NVM, the polarization state of the ferroelectric gate dielectric layer is switched by the supplying programming (P) and erasing (E) gate voltages, which control the channel current (*I*_*DS*_) between the drain and source electrodes. Ferroelectric polymer is a desired material to construct Fe-OFET NVMs due to its many advantages, such as, mechanical flexibility, low-cost, easy solution processability and low-temperature fabrication^1–5^. In the last decade, great efforts have been devoted to develop and improve the performances of Fe-OFET NVMs and to develop flexible memories with ferroelectric poly(vinylidene fluoride-trifluoreoethylene) [P(VDF-TrFE)] as gate dielectric, attributing to its spontaneous formation of ferroelectric *β* phase polycrystal and large remanent polarization^1–19^.

The bottleneck issue, high operating voltage in Fe-OFET NVMs, must be resolved before considering its commercial application. High operating voltages, often ranging from 30 to 100 V, were universally needed to switch the memory states in previous reports, due to the high coercive field (*E*_*C*_, up to 50 MV m^−1^) and the large film thickness of ferroelectric P(VDF-TrFE)^1, 3–13^. The low operating voltages of 8 ~ 18 V have been demonstrated in a few reported Fe-OFET NVMs with a thin P(VDF-TrFE) film (about 100 nm)^14–17^. However, the operating voltage of 8 V is not low enough to satisfy the requirements of the practical applications, not mention to the side effects that gate leakage current became severe with the decreasing thickness of P(VDF-TrFE) film, which weakened the reliability and stability of memories^17, 18^. Some extra issues also occurred, such as reduction in polarization and increase of switching field^20–22^. Thus, the reduction of the operating voltage in Fe-OFET NVMs is limited through purely decreasing the thickness of ferroelectric P(VDF-TrFE) film. Similar to the method that employing the insulator materials with a high dielectric constant as the gate dielectric layers can reduce the operating voltage in the conventional OFETs^23, 24^, employing a ferroelectric polymer with an enough low *E*_*C*_ as the gate dielectric, such as terpolymer poly(vinylidene-fluoride-trifluoroethylene-chlorotrifluoroethylene) [P(VDF-TrFE-CTFE)], is an alternative route to reduce the operating voltage of Fe-OFET NVMs. However, a relative high operating voltage was needed to switch the memory states due to the thick ferroelectric P(VDF-TrFE-CTFE) gate dielectric that was required to minimize the gate leakage current in the last a few reports^25^. Additionally, the method that employing a reasonable interfacial layer to improve the mobility and to reduce the leakage current in conventional OFETs should also be applicable to Fe-OFETs^8, 24, 26^. Combining two concepts of “low *E*_*C*_ ferroelectric polymer” and “ultrathin ferroelectric film” is a desirable route to achieve a Fe-OFET NVM operating at the satisfactory low voltage, preferably ≤5 V, which has not been reported.

In this work, we propose a novel route to resolve the above bottleneck issue, by employing two ultrathin AlO_X_ interfacial layers sandwiching an ultrathin ferroelectric polymer gate dielectric with a low *E*_*C*_, in the flexible Fe-OFET NVM. Excellent memory performances, with large mobility, high reliable memory switching endurance and stable memory retention capability, are achieved at the low operating voltage of 4 V, which is lowest value reported to data for all Fe-OFET NVMs. Moreover, outstanding mechanical fatigue property is also demonstrated in our flexible memory.

## Experimental

On the 125-μm thick polyethylene naphthalate (PEN) substrates, a 40 nm thick Al film was thermally deposited as gate electrodes. On the surface of Al gate electrodes, the ultraviolet-ozone (UVO) treatment was performed at a power of 28 mW cm^−2^ for 20 min to obtain an ultrathin AlO_X_ interfacial layer^27–30^. Ferroelectric polymer P(VDF-TrFE-CTFE) (composition of 64.2/27.1/8.7 mol%, purchased from Piezotech-Arkema Corp., France.) was spin-coated on the AlO_X_ covered gate electrodes from the solutions in butyl acetate, annealing at 120 °C for 120 min to remove the residual solvent. The thicknesses of P(VDF-TrFE-CTFE) films were measured by a XP-2 Stylus surface profiler, which depended on the concentration of the solutions. Following, an 4 nm thick Al film was thermally evaporated on the surfaces of P(VDF-TrFE-CTFE) films at a very slow evaporating rate of about 0.1 ~ 0.2 Å s^−1^. The evaporating rate and thickness of the Al film was carefully controlled by a quartz crystal thickness monitor. Next, the Al film was completely oxidized by UVO treatment for 30 min to obtain an ultrathin AlO_X_ interfacial layer^27–30^. Finally, 40 nm thick pentacene film and bilayer source-drain electrodes consisting of MoO_3_ (8 nm) and Cu (60 nm) were thermally deposited in sequence at the rate of 0.2, 0.2 and 1.0 Å s^−1^, respectively, and were patterned by corresponding shadow masks. The channel length (*L*) and width (*W*) were 100 and 1000 µm, respectively. Additional, capacitors with structures of Al/AlO_X_ (with or without) /P(VDF-TrFE-CTFE)/AlO_X_ (with or without) /Cu were also prepared at the same conditions with the Fe-OFET NVMs. The electrical properties of the FE-OFET NVMs and the capacitances were measured by a semiconductor parameter analyzer (Agilent B1500A) in the ambient atmosphere at room temperature. The surface morphologies of P(VDF-TrFE-CTFE), AlO_X_ and pentacene films were characterized by tapping-mode atomic force microscopy (AFM) (Dimension Icon, Bruker Co.).

## Results and Discussion

**Figure 1:**
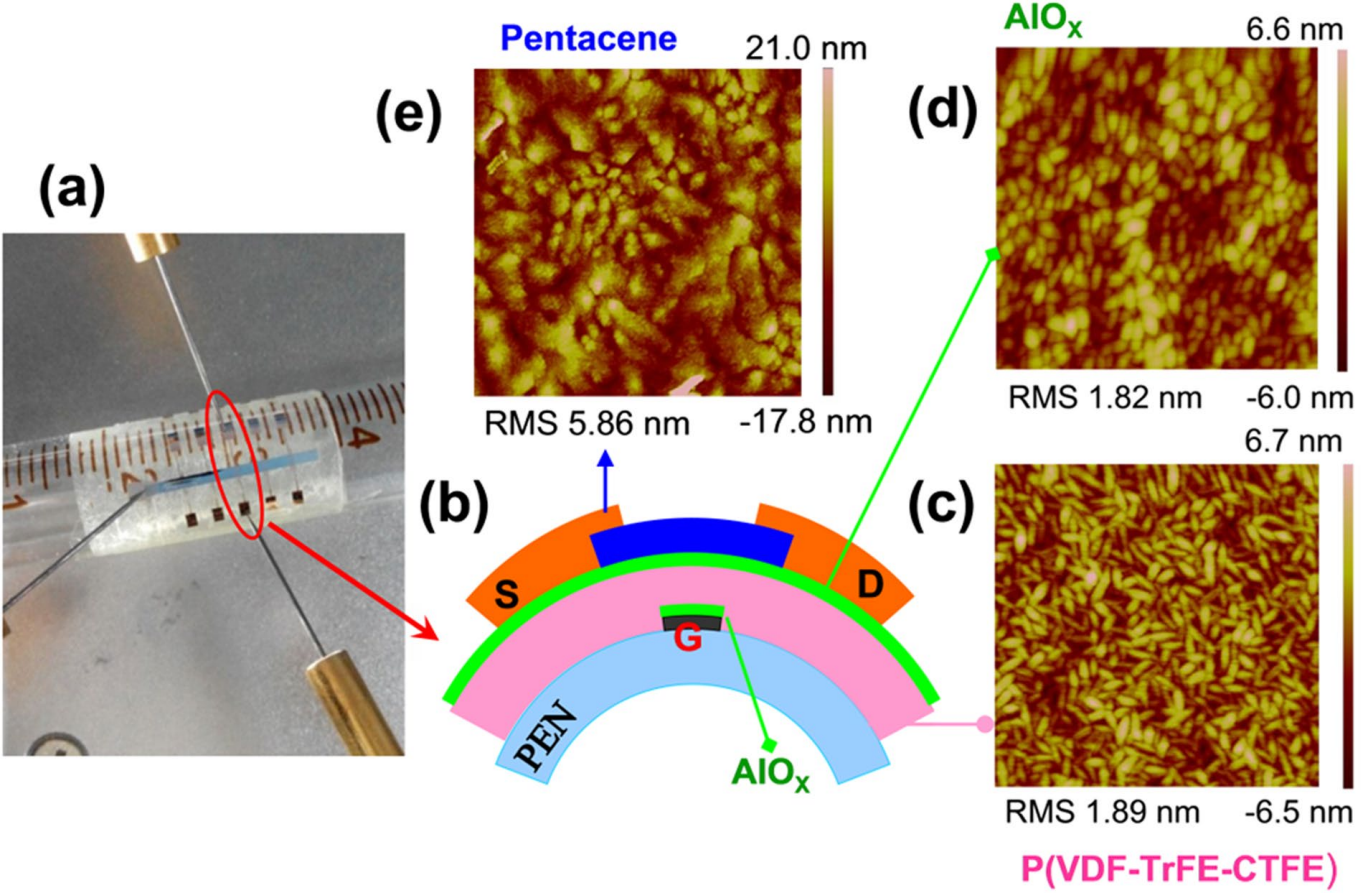
(**a**) Photograph and (**b**) schematic configuration of the flexible Fe-OFET NVM. AFM images of (**c**) 60 nm thick P(VDF-TrFE-CTFE) film (2 μm × 2 μm), (**d**) AlO_X_ interfacial layer (2 μm × 2 μm) and (**e**) pentacene film (5 μm × 5 μm).

Figure 1a and b show the photograph and schematic configuration of our fabricated flexible Fe-OFET NVMs. After the UVO treatment on the Al gate electrode, an ultrathin AlO_X_ interfacial layer was formed for reducing the gate leakage current, as demonstrated in the following. On the ultrathin AlO_X_ interfacial layer coated Al gate electrode, the spin-coated P(VDF-TrFE-CTFE) films, with the thickness downscaling to 205, 150, 100, 60 and 40 nm, respectively, exhibited similar microstructures and surface morphologies with many rod-like crystalline grains, as shown in Figs 1c and S1 (Supporting Information). The average grain size was about 160 and 60 nm in length and width, respectively. The surface root-mean-square (RMS) roughness of these P(VDF-TrFE-CTFE) films was less than 1.9 nm. Our previous work demonstrated that the ultrathin AlO_X_ film, processed by present technology on the polymer poly(methyl methacrylate) (PMMA), possessed a uniform thickness^31^. Here, these ultrathin AlO_X_ interfacial layers on the surfaces of different thick ferroelectric films exhibited an ellipse-like grain morphologies, which approximately retraced the polycrystalline morphologies of the underlying P(VDF-TrFE-CTFE) films, as demonstrated by the AFM image in Fig. 1d, indicating that they also possessed a uniform thickness. On the AlO_X_ interfacial layers, the deposited pentacene films exhibited good polycrystalline morphologies with an average RMS value of 5.86 nm and an average grain size of about 500 nm × 750 nm, as shown in Fig. 1e.

**Figure 2:**
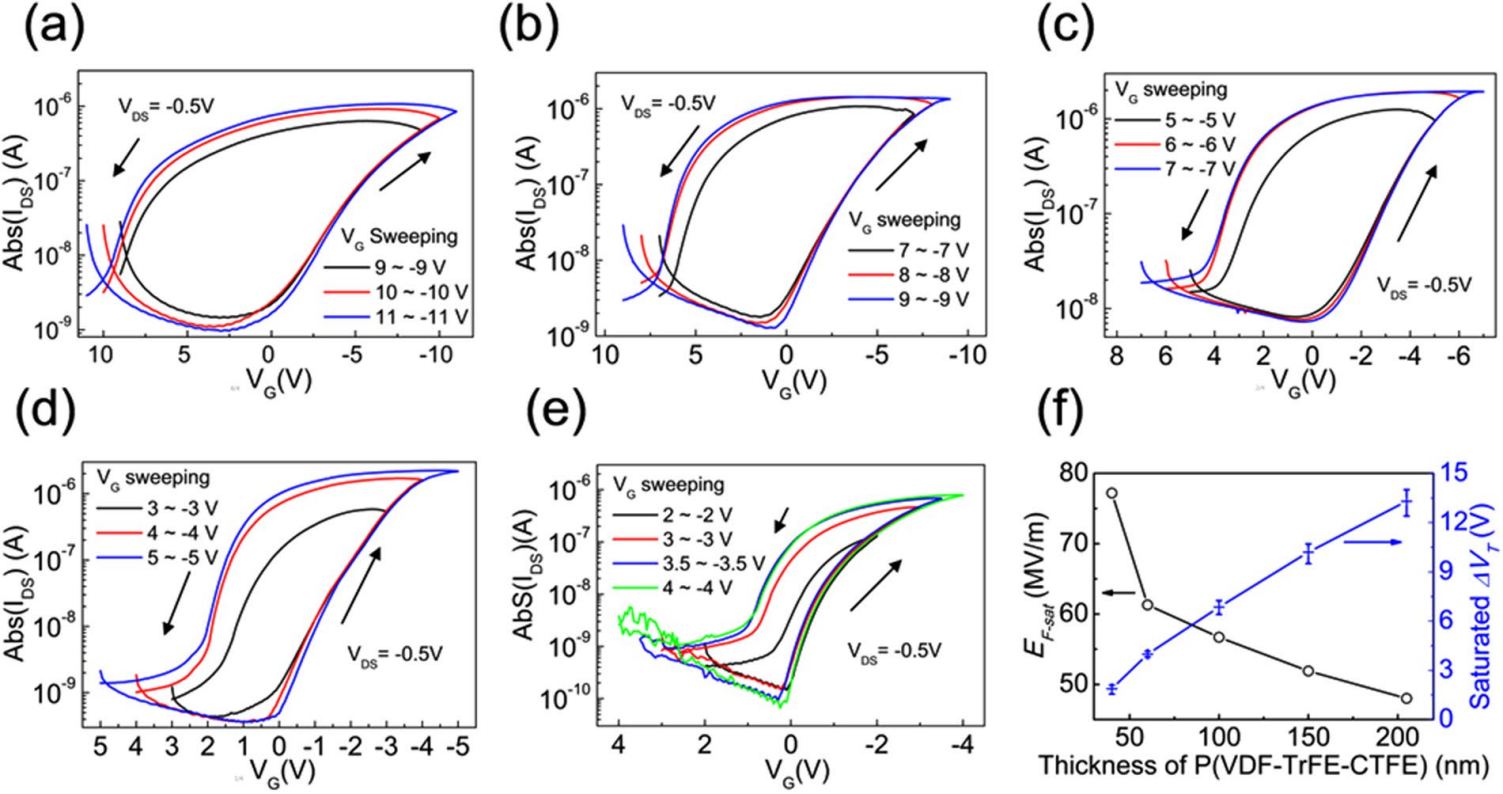
Transfer characteristics of the Fe-OFET NVMs with a downscaling P(VDF-TrFE-CTFE) film at different thicknesses of (**a**) 205, (**b**) 150, (**c**) 100, (**d**) 60 and (**e**) 40 nm, respectively, operating at different *V*_*G*_ sweeping ranges. (**f**) Dependence of the saturated *ΔV*_*T*_ and the corresponding electric field (*E*_*F-sat*_) on the thickness of P(VDF-TrFE-CTFE) films.

Figures 2 and S2 show the transfer characteristics of the flexible Fe-OFET NVMs with two ultrathin AlO_X_ interfacial layers sandwiching different thick ferroelectric P(VDF-TrFE-CTFE) films, in exponential and linear coordinates, respectively, operating in the linear region with drain-source voltage (*V*_*DS*_) of −0.5 V. For all devices, prominent anticlockwise hysteresis properties were obtained at the supplied bidirectional sweeping gate voltage (*V*_*G*_). The hysteresis mechanism was attributed to the ferroelectric property of polymer P(VDF-TrFE-CTFE), demonstrated by the P-E hysteresis loops obtained from the capacitors with the structures of Al/AlO_X_ (with or without)/P(VDF-TrFE-CTFE) (60 nm)/AlO_X_ (with or without)/Cu (Fig. S3). The *E*_*C*_ of these ferroelectric layers was about 21.6 V μm^−1^, and was hardly affected by the AlO_X_ interfacial layer. The remanent polarization (*P*_*r*_) value of a pure P(VDF-TrFE-CTFE) film was about 5.0 mC m^−2^ at the supplied electric filed of 167 V/μm, and the *P*_*r*_ had a slightly increase when the AlO_X_ interfacial layers were employed. The low *E*_*C*_ suggests a promising application of P(VDF-TrFE-CTFE) in pursuing the low-voltage operating FE-OFET NVMs. Analogous to the conventional field-effect transistor, the linear mobility (*μ*) can be extracted from the transfer characteristics according to,

1 $${I}_{DS}=\frac{W}{L}\mu {C}_{i}({V}_{G}-{V}_{T}-\frac{{V}_{DS}}{2}){V}_{DS},$$

where, *V*_*T*_ and *C*_*i*_ are threshold voltage and gate dielectric layer capacitance per unit area, respectively. The values of *C*_*i*_ were extracted based on the measurement on capacitors, as shown in Fig. S4. High mobilities of 1.7 ~ 3.3 cm^2^ V^−1^ s^−1^ were achieved in our Fe-OFETs, independent on both *V*_*G*_ sweeping ranges and thickness of P(VDF-TrFE-CTFE) films.

In all Fe-OFETs, the hysteresis loops increased and reached saturation with the enlargement of the bidirectional *V*_*G*_ sweeping ranges, as shown in Fig. 2a–e and Fig. S2. The sweeping range of *V*_*G*_, required for achieving a saturated hysteresis loop, were ±10, ±8, ±6, ±4 and ±3.5 V in these Fe-OFETs with the thickness of P(VDF-TrFE-CTFE) films downscaling to 205, 150, 100, 60 and 40 nm, respectively. The hysteresis in the *I*_*DS*_ - *V*_*G*_ curve allows us to define the binary 1 and 0 states by determining the *V*_*T*_ or the *I*_*DS*_ at reading gate voltage (*V*_*R*_ = *V*_*G*_) of 0 V. The memory window (*ΔV*_*T*_) is defined as the difference of both *V*_*T*_ at the 1 and 0 states, respectively. The saturated *ΔV*_*T*_, extracted from the saturated hysteresis loops, exhibited an obvious dependence on the thickness of P(VDF-TrFE-CTFE) films, as summarized in Fig. 2f. The average *ΔV*_*T*_, extracted from ten devices on one substrate, were 13.3, 10.2, 6.85, 3.98 and 1.88 V, respectively. Comparing these Fe-OFETs, the reducing degree of the *V*_*G*_ to achieve a saturated *ΔV*_*T*_ obviously declined with the further downscaling P(VDF-TrFE-CTFE) film thickness from 60 to 40 nm. One of the reasons was attributed to the employment of two AlO_X_ interfacial layers, which shared larger proportion of the supplied *V*_*G*_ with the downscaling thickness of P(VDF-TrFE-CTFE) films. The data suggested that the decreasing degree of the operating voltage in FG-OFET NVMs should be limited by purely downscaling the thickness of ferroelectric P(VDF-TrFE-CTFE) film. To further understand the reasons of this limitation, the electric field (*E*_*F-Sat*_), supplied on the ferroelectric films for achieving saturated *ΔV*_*T*_, was summarized as a function of their thickness, as shown in Fig. 2f. Here, the *E*_*F-Sat*_ was carefully calculated from the series capacitors consisting of two ultrathin AlO_X_ layers and P(VDF-TrFE-CTFE) film, from the actually measured capacitances (Fig. S4a). In our Fe-OFETs, the *E*_*F-Sat*_ increased at a approximately linearly relation with the downscaling P(VDF-TrFE-CTFE) film thicknesses from 205 to 60 nm (Fig. 2f). However, a sharp increase of the *E*_*F-Sat*_ was observed when the thicknesses of P(VDF-TrFE-CTFE) films were further downscaled from 60 to 40 nm (Fig. 2f). The increasing *E*_*F-Sat*_ indicated an increasing switching field with the downscaling P(VDF-TrFE-CTFE) film thickness, especially in the case of tens of nm thickness, which was another important reason to limit the further decrease of the operating voltage in Fe-OFETs. The similar result has been demonstrated on ferroelectric P(VDF-TrFE) film in previous reports^20, 21^.

Although, obvious hysteresis loops with *ΔV*_*T*_ of about 2.7 and 1.6 V were obtained at the bidirectional *V*_*G*_ sweeping range of ±3.0 V in the Fe-OFETs with a 60 and 40 nm thick P(VDF-TrFE-CTFE) film, respectively. The voltages of ±3.0 V should be close to the limited values for P/E operations in our Fe-OFET NVMs. Considering the practical application, the enough large *ΔV*_*T*_ and memory on-off ratio are desired for avoiding reading mistake and prolonging the data storage retention time. Thus, the Fe-OFET with a 60 nm thick P(VDF-TrFE-CTFE) film was considered as the optimal device for the pursuit of the low-voltage operating memory, in which the enough large saturated *ΔV*_*T*_ of about 4.0 V and memory on-off ratio of 10^3^ were achieved at the operating voltage of 4 V, which is the lowest value reported to data for all Fe-OFET NVMs.

**Figure 3:**
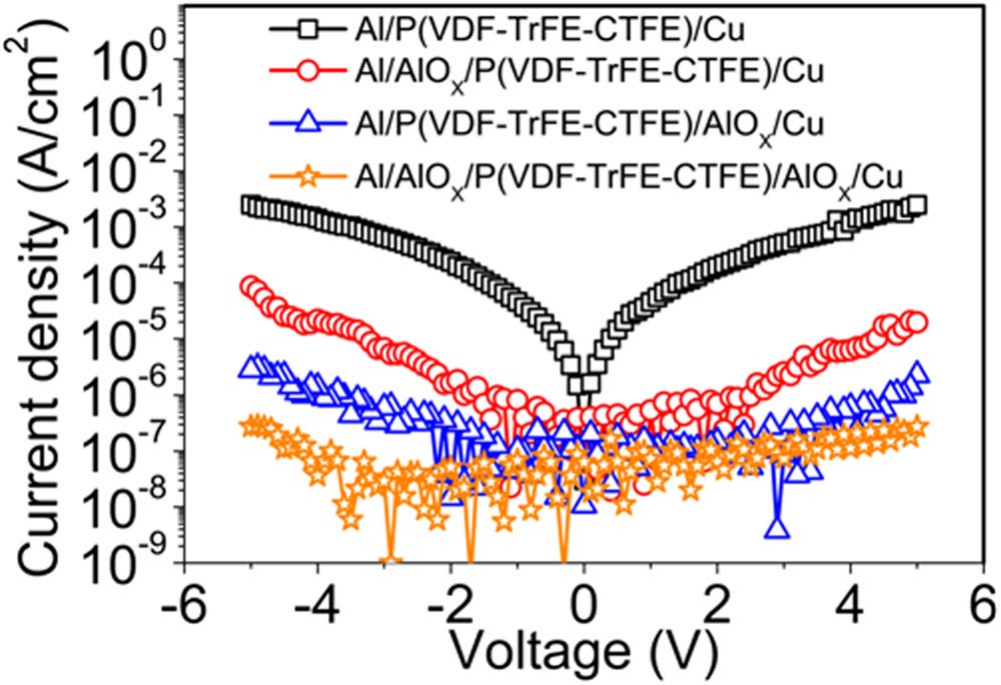
The leakage current density versus voltage of the capacitors with/without ultrathin AlO_X_ layers at each side of the 60 nm thick P(VDF-TrFE-CTFE) film.

The gate leakage current is another important factor to prevent the realization of the low-voltage operating Fe-OFET NVMs since it become worse with the decreasing thickness of ferroelectric gate layer, which degrades the reliability and stability of the memory. Here, we employed two ultrathin AlO_X_ interfacial layers to minimize the gate leakage current in Fe-OFETs. The ultrathin interfacial layer with a higher dielectric constant (such as AlO_X_) favors to share less gate field and thus favor to reduce P/E voltages in Fe-OFET NVMs. In the capacitor with a single 60 nm thick P(VDF-TrFE-CTFE) film, the leakage current density was visible with 10^−3^ A cm^−2^ at the voltages of ±5 V, as shown in Fig. 3. But, the leakage current density was reduced by 1 and 3 orders of magnitude after an ultrathin AlO_X_ layer was prepared at the interfaces of gate electrode/P(VDF-TrFE-CTFE) and P(VDF-TrFE-CTFE)/pentacene, respectively (Fig. 3). When two ultrathin AlO_X_ interfacial layers were employed at both sides of the P(VDF-TrFE-CTFE) film, the leakage current density was further decreased to 10^−7^ A cm^−2^ at the voltages ±5 V (Fig. 3), which is small enough for the reliable and stable operation of Fe-OFET NVMs. In addition, the ultrathin AlO_X_ interfacial layer between pentacene and P(VDF-TrFE-CTFE) films played another important role to enhance mobility, as demonstrated by the transfer characteristics of the reference Fe-OFETs in Fig. S5. Both the on current and mobility of the Fe-OFET with an ultrathin AlO_X_ layer at the interface of pentacene/P(VDF-TrFE-CTFE) were improved over 3 orders of magnitude compared to those of the Fe-OFET without an AlO_X_ layer (Fig. S5b). The reasons for the enhancement of mobility were attributed to the better pentacene morphology deposited on AlO_X_ layer than that on P(VDF-TrFE-CTFE) (Figs 1e and S5c) and the suppression of the polarization fluctuation from the orientation difference of dipole moments among ferroelectric microcrystals, which was demonstrated to degrade the mobility of FE-OFETs^8^. In additional, the bilayer source-drain electrodes consisting of MoO_3_/Cu possessed a very good ohm contact, which was beneficial for achieving high mobility^32^.

**Figure 4:**
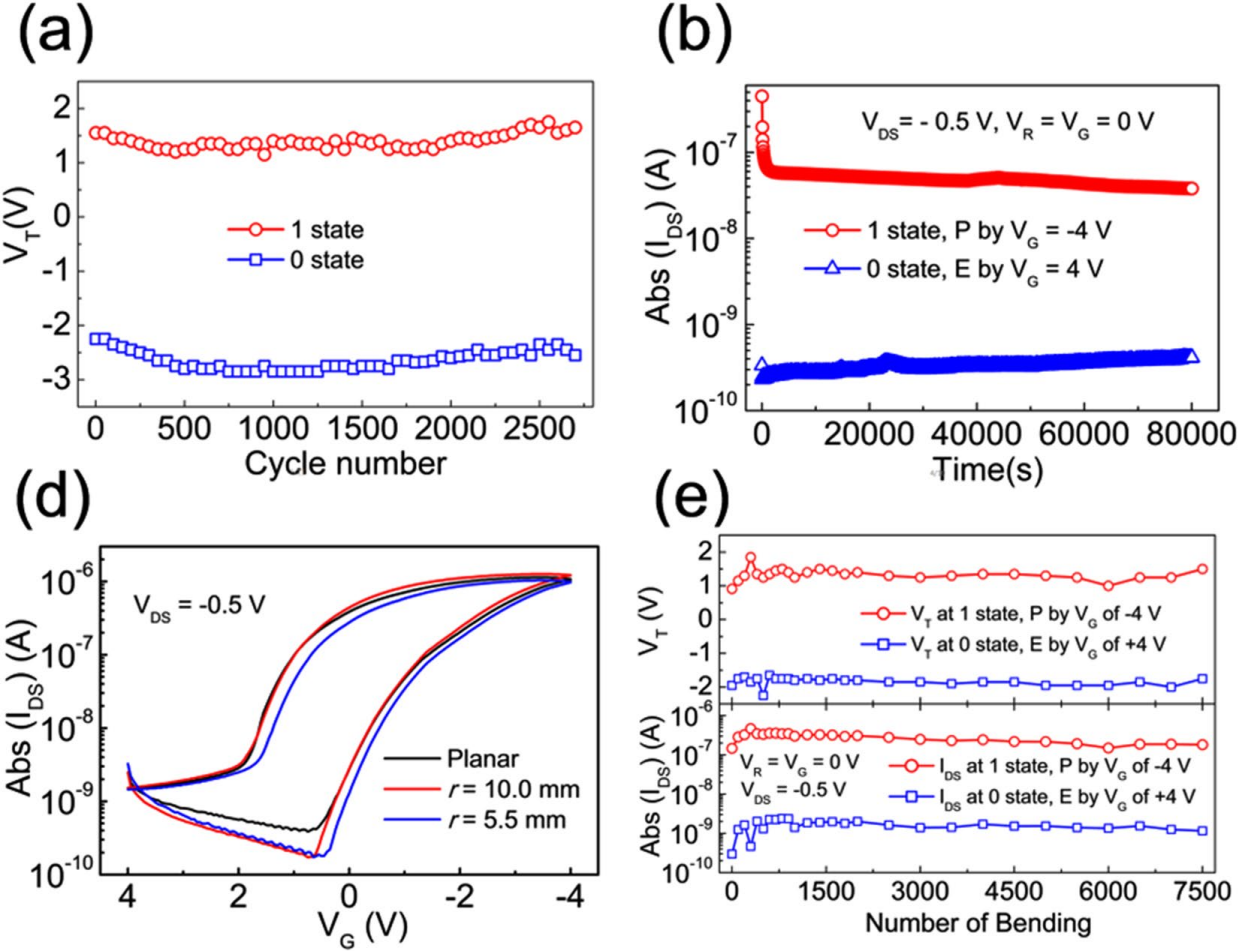
(**a**) Memory switching endurance and (**b**) data storage retention characteristics of the low-voltage operating flexible Fe-OFET NVM with a 60 nm thick P(VDF-TrFE-CTFE) ferroelectric film. (**c**) Transfer characteristics of the low-voltage operating flexible Fe-OFET NVM measured at the planar and bending states, respectively. (**d**) Mechanical fatigue property of the low-voltage operating flexible Fe-OFET NVM measured at a bending radius of 5.5 mm.

Figure 4a shows the memory switching endurance properties of the optimal low-voltage operating flexible Fe-OFET NVM. At the supplied circular P/E voltages (*V*_*G*_) of ±4 V, the programmed and erased *V*_*T*_ was switched between 1 and 0 states. Both *V*_*T*_ at 1 and 0 states maintained well with a slight fluctuation over 2700 cycles (Fig. 4a), indicating an outstanding operating reliability. The data storage retention capability was investigated by monitoring the reading *I*_*DS*_ at both 1 and 0 states as a function of time, after the P/E operations at ±4 V, respectively, as shown in Fig. 4b. The reading state was defined as *V*_*DS*_ of −0.5 V and *V*_*R*_ of 0 V. A rapid degradation of the reading *I*_*DS*_ at 1 state was observed in the initial period of 600 s due to the release of the charges until the equilibrium reached. Then, the reading *I*_*DS*_ at both 1 and 0 states maintained well with a neglectable degradation and the memory on-off ratio always larger than 10^2^ during the measuring time of 8 × 10^4^ s (Fig. 4b), indicating a high stable data storage retention capability. As a result, the whole performance parameters of our flexible memory were comparable to or even superior to those reported in other state-of-art Fe-OFET NVMs and inorganic semiconductor based ferroelectric transistor NVM^5, 25, 33, 34^, as summarized in Table 1 (Supporting Information).

The mechanical fatigue property is also important for the practical application of a flexible memory. Figure 4c shows the hysteresis characteristics of a low-voltage operating flexible Fe-OFET memory measured at the planar and stretching bending states with the bending radius (*r*) of 10.0 and 5.5 mm, respectively. In the case of *r* = 10.0 mm, the hysteresis loop exhibited a good repeatability, compared with that measured at the planar state. With the *r* decrease to 5.5 mm, the hysteresis loop of the memory exhibited a slight positive shift, compared with those in the other two cases, due to the larger stretching bending channel. The mechanical fatigue property was further investigated by measuring the memory properties at the planar state, after the substrate was repeatedly bended at *r* = 5.5 mm for 100, 200 and 500 times, respectively. Figure 4d shows the measured results with the *V*_*T*_ and the reading *I*_*DS*_ at both 1 and 0 states as a function of the bending number. Both *V*_*T*_ and reading *I*_*DS*_ at both 1 and 0 states exhibited excellent repeatability with a slight fluctuation over 7500 cycles, indicating that our low-voltage operating flexible Fe-OFET NVM possessed an excellent mechanical fatigue property.

## Conclusions

In summary, we have demonstrated an record low-voltage operating flexible Fe-OFET NVM by employing two ultrathin AlO_X_ interfacial layers sandwiching an ultrathin ferroelectric P(VDF-TrFE-CTFE) film with a low coercive field. In the fabricated flexible Fe-OFET NVM, excellent memory performances, with large mobility of 1.7 ~ 3.3 cm^2^ V^−1^ s^−1^, high reliable memory switching endurance over 2700 cycles and stable data storage retention capability over 8 × 10^4^ s with memory on-off ratio larger than 10^2^, were achieved at low P/E voltages of ±4 V. Simultaneously, outstanding mechanical fatigue property with the memory performances maintaining well over 7500 bending cycles at *r* of 0.55 mm was also achieved in the flexible Fe-OFET NVM.

## Electronic supplementary material

Supplementary information
