# Supplementary material for: Excellent low-voltage operating flexible ferroelectric organic transistor nonvolatile memory with a sandwiching ultrathin ferroelectric film
Source: Sci Rep. 2017 Aug 21;7:8890. doi: 10.1038/s41598-017-09533-2 (PMC5566424; doi:10.1038/s41598-017-09533-2)
Supplement: Supplementary file 1 — Supplementary information [file 41598_2017_9533_MOESM1_ESM.doc]

Supplementary Information

**Excellent low-voltage operating flexible ferroelectric organic transistor nonvolatile memory with a sandwiching ultrathin ferroelectric film**

Ting Xu,^†^ Lanyi Xiang,^†^ Meili Xu, Wenfa Xie, and Wei Wang*

State Key Laboratory on Integrated Optoelectronics, College of Electronic Science and Engineering, Jilin University, 2699 Qianjin Street, Changchun 130012, China.

^†^ These authors contributed equally to this work.

^*^ Correspondence should be addressed to Wei Wang (email: wwei99@jlu.edu.cn)


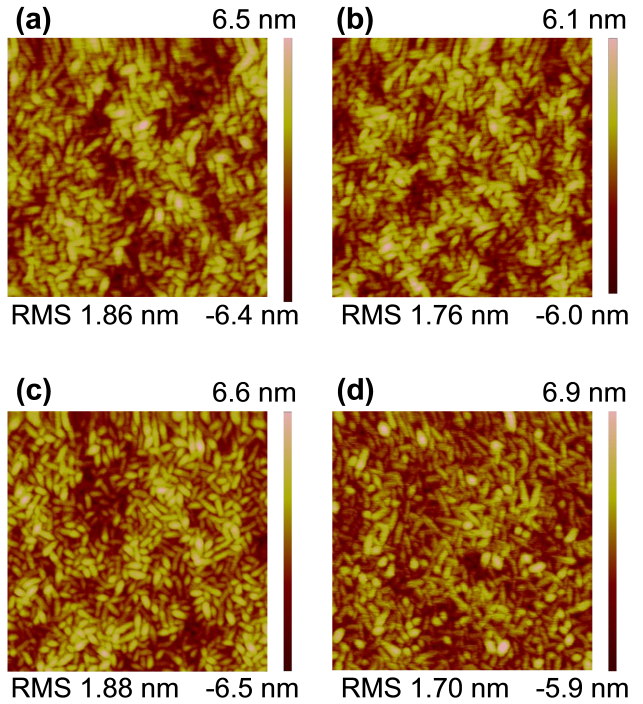


**Figure S1.** AFM images of the P(VDF-TrFE-CTFE) films with the thicknesses of (a) 205, (b) 150, (c) 100, and (d) 40 nm, respectively, spin-coated on the ultrathin AlO_X_ interfacial layer coated Al gate electrodes. The size area is of 2 μm ×2 μm.


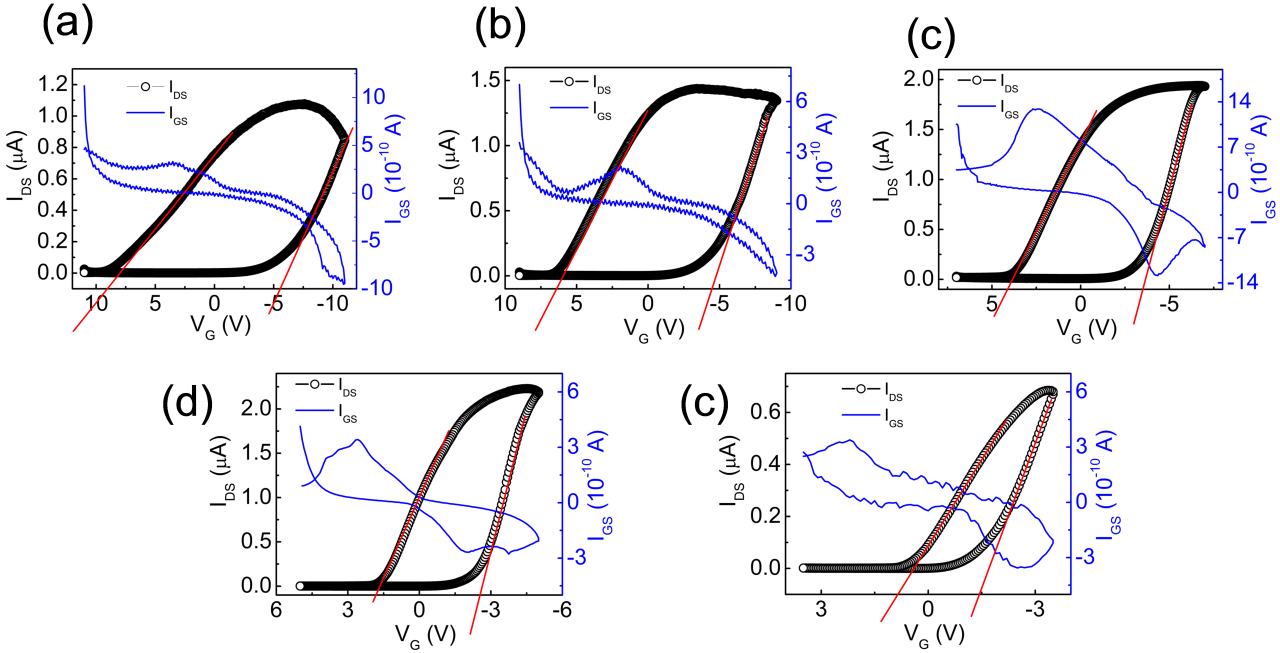


**Figure S2.** Transfer characteristics of the Fe-OFET NVMs with a downscaling P(VDF-TrFE-CTFE) film at different thicknesses of (a) 205, (b) 150, (c) 100, (d) 60 and (e) 40 nm, respectively, operating at different *V_G_* sweeping ranges.


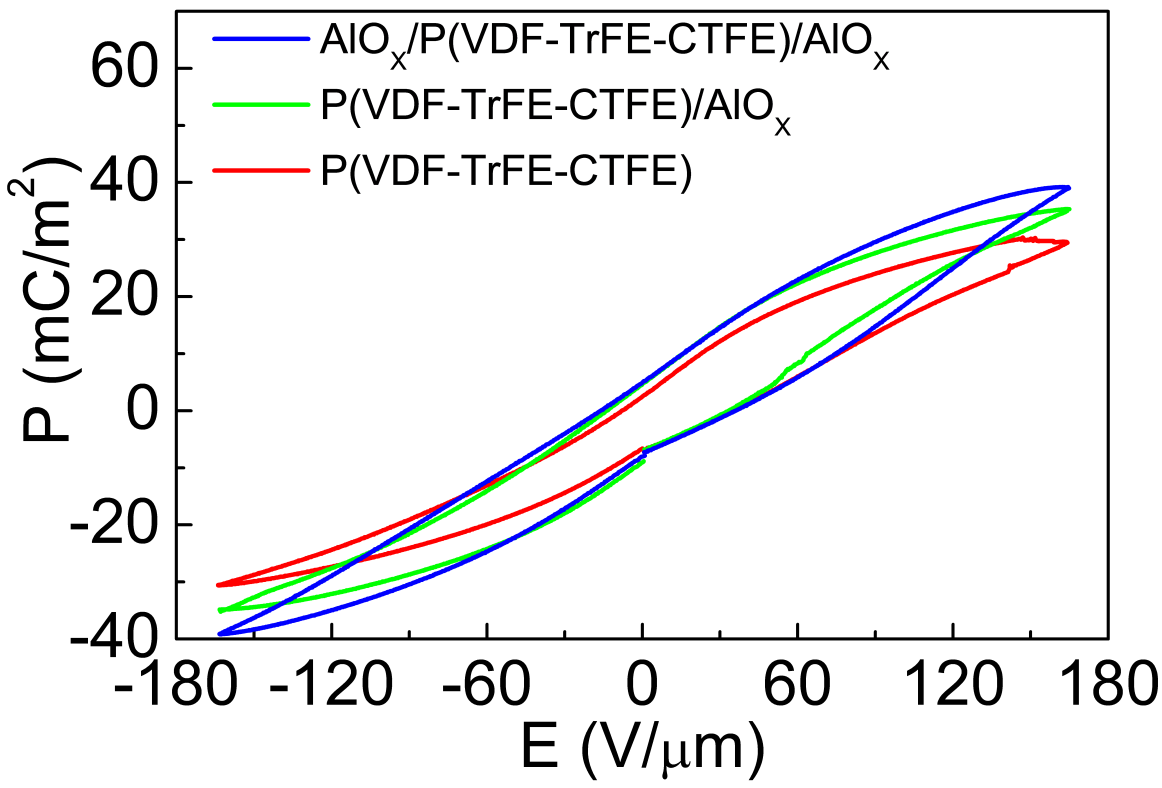


**Figure S3.** The polarization-electric filed (P-E) hysteresis property of the 60 nm thick P(VDF-TrFE-CTFE) films with or without ultrathin AlO_X_ interfacial layers, measured by P-PMF1213-346 ferroelectric test system (Radiant Technology, U.S.A.).


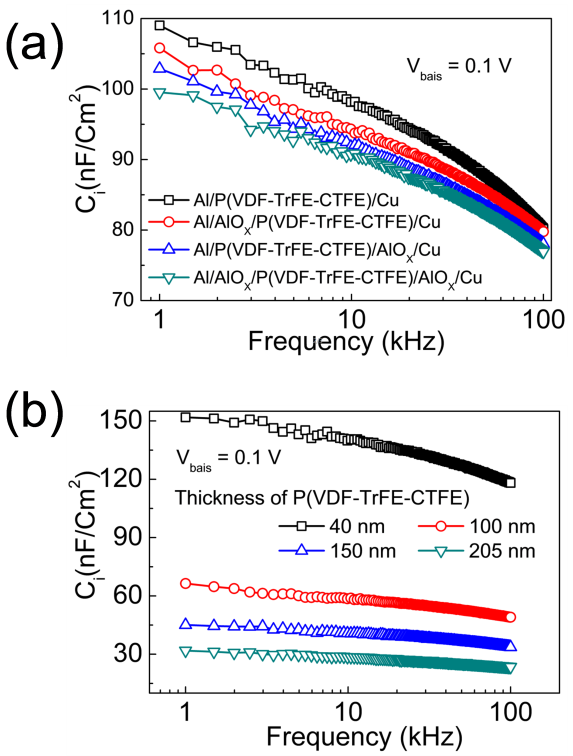


**Figure S4.** (a) C-F characteristics of the capacitors with/without ultrathin AlO_X_ layer at each side of the 60 nm thick P(VDF-TrFE-CTFE) film. (b) C-F characteristics of the capacitors with a structure of Al/ AlO_X_/P(VDF-TrFE-CTFE)/AlO_X_/Cu with different P(VDF-TrFE-CTFE) film thicknesses.


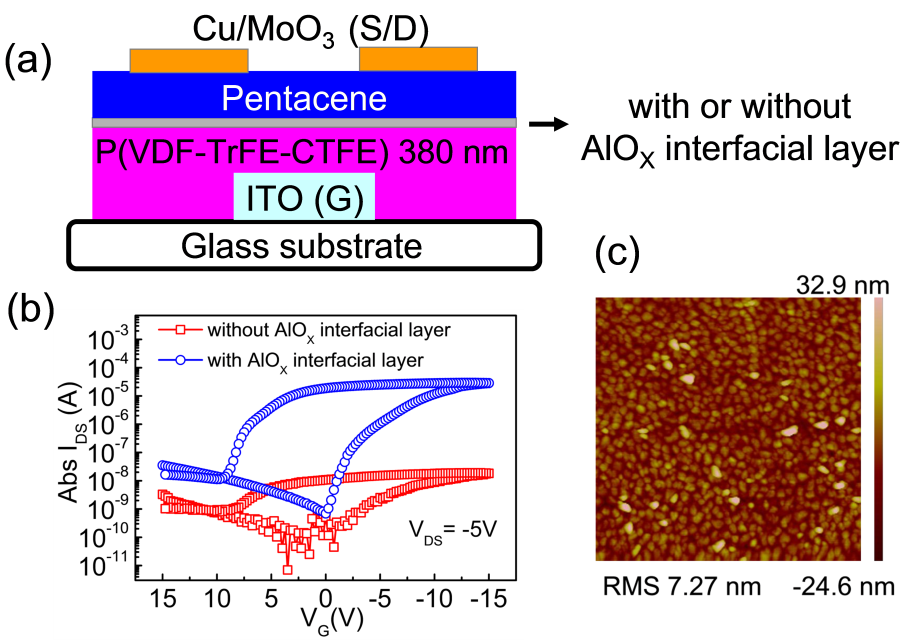


**Figure S5.** (a) Schematic configuration of the reference FE-OFETs. (b) The transfer characteristics of the reference FE-OFETs with or without an ultrathin AlO_X_ layer at the interface of pentacene/P(VDF-TrFE-CTFE) films. (c) AFM image of a 40 nm thick pentacene film deposited on the surface of P(VDF-TrFE-CTFE) film (size: 5 μm ×5 μm). The mobility of the Fe-OFET with an AlO_X_ interfacial layer was about 2.7 cm^2^ V^-1^ S^-1^. However, the mobility of the Fe-OFET without AlO_X_ interfacial layer was about 1.5 × 10^-3^ cm^2^ V^-1^ S^-1^.

**Table 1.** The performances comparison of our Fe-OFET NVM with previous reported Fe-OFET NVMs and some inorganic semiconductor based ferroelectric NVMs.

| Substrate | Semi-conductor | Mobility  [cm^2^ V^-1^ s^-1^] | Operating voltage [V] | Endurance  [cycle number] | Retention time [s] | Refs. |
| --- | --- | --- | --- | --- | --- | --- |
| Rigid | MEH-PPV | 1.3 × 10^-3^  ~ 2.4 × 10^-4^ | > 77.5 | 1000 | 10,000 | 1 |
|  | PTAA | / | 100 | /^a)^ | 40,000 | 11 |
|  | Pentacene | 0.49 | 40 | / | 4,500 | 9 |
|  | C_8_-BTBT | 4.6 | 30 | / | 4,000 | 8 |
|  | P3HT | 0.1 | 15 | / | 10,000 | 17 |
|  | P3HT | 8.0 × 10^-3^ | 15 | / | 10,000 | 15 |
|  | TIPS-PEN | 0.65 | 15 | / | 50,000 | 14 |
|  | TIPS-PEN | / | 8 | / | 6,000 | 16 |
|  | InGaZnO_4_ | / | 40 | / | 2,700 | 25 |
|  | ZnO nanowire | 67 | 20 | / | 10,000 | 33 |
| Flexible | Pentacene | 0.1 ~ 0.18 | 13 ~ 20 | / | 10,000 | 19 |
|  | Pentacene | 0.6 | 60 | 10^4^ ^b)^ | 5 months ^b)^ | 10 |
|  | P3HT | / | 80 | 120 | 10,000 | 12 |
|  | QQT(CN)4 | < 0.1 | 50 | 100 | 7,000 | 13 |
|  | F8T2 | 5.0 × 10^-2^ | 20 | / | 2,000 | 6 |
|  | Pentacene | / | 30 | 2500 | 1 month | 4 |
|  | SnO | 3.3 | 30 | 200 | 5,000 | 5 |
|  | IGZO | 21 | 20 | / | 3,500 | 34 |
|  | Pentacene | 1.7 ~ 3.3 | 4 | 2700 | 80,000 | This work |

^a)^ The corresponding data were not reported in these references.

^b)^ The data were obtained from a Fe-OFET NVM with an organic/metallic encapsulation layer. But, the endurance number and retention time of the Fe-OFET NVM without encapsulation layer were 400 cycles and 10^4^ s, respectively.
